# The Influence of Horsetail (*Equisetum arvense* L.) Powder and Horsetail-Based Silica on the Crystallization Kinetics of Polylactide

**DOI:** 10.3390/ma17235697

**Published:** 2024-11-21

**Authors:** Olga Mysiukiewicz, Joanna Szulc, Andrzej Miklaszewski

**Affiliations:** 1 Institute of Materials Technology, Poznan University of Technology, Piotrowo 3, 61-138 Poznan, Poland; 2 Department of Food Industry Technology and Engineering, Bydgoszcz University of Science and Technology, Seminaryjna 3, 85-326 Bydgoszcz, Poland; joanna.szulc@pbs.edu.pl; 3 Institute of Materials Science and Engineering, Poznan University of Technology, Jana Pawla II 24, 61-138 Poznan, Poland; andrzej.miklaszewski@put.poznan.pl

**Keywords:** polylactide, crystallization, silica, horsetail

## Abstract

Biogenic silica (SiO_2_) sourced from living organisms, especially plants such as rice and other cereals, has recently been successfully applied in different polymeric compositions. Another rich source of biogenic silica is common horsetail (*Equisetum arvense* L.), containing up to 25% SiO_2_ in the dry matter. In this study, biogenic silica was obtained from horsetail powder by acid leaching in sulfuric acid and calcination at 400 °C. The analysis, including measurements of specific surface area using the Brunauer–Emmett–Teller method, assessment of crystallinity by X-ray diffraction, as well as chemical content analysis by Fourier-transform infrared spectroscopy showed that high-purity, high-surface mesoporous silica was obtained. The biogenic silica and horsetail powders were also introduced to polylactide (PLA) to determine their influence on the polymer’s crystallization, which was studied in both non-isothermal and isothermal conditions by differential scanning calorimetry. The crystallization parameters were calculated according to the Avrami method based on isothermal crystallization curves at 100, 110 and 120 °C. The crystalline structures were observed by optical microscopy in polarized light. It was found that both fillers improve the crystallization of PLA, especially in low-supercooling conditions, so they can be successfully utilized in industrial applications, when high crystallinity of polylactide is needed.

## 1. Introduction

Silicon is one of the most abundant elements present in the Earth’s crust [1]. It is a prominent component of soils, where it can be found in form of silica (SiO_2_) or salts of silicic acid (silicates) [2]. Si-bearing minerals such as quartz, chalcedony, opal, or different clays undergo weathering, which results in the creation of aqueous monosilicic acid (H_4_SiO_4_) [3]. This compound is then absorbed by plants and undergoes polymerization in their tissues, creating a form of porous opal (SiO_2_·nH_2_O) called phytoliths [1]. Even though Si is not an essential element for plants [3], silica contained in plant tissues improves their mechanical strength as well as their resistance to diseases and other disadvantageous external factors [4]. The concentration of silica in plants depends on the species, and the Poaceae/Gramineae and Equisetaceae families are widely known for their SiO_2_-acumulating properties, as content of silica in their ash may be as high as 50–70% [5]. This compound can be relatively easily sourced from the plant matter and there are numerous scientific articles describing preparation, properties, and applications of biogenic silica originating mainly from straw and husks sourced from rice [5,6,7] and other crops, including wheat, sugarcane, oil palm, or barley [8]. Plant-based SiO_2_ can be a more environmentally friendly alternative to silica precipitated using the conventional method from sodium silicate, which not only is very energy- and water-consuming but also results in production of large amounts of hazardous by-products such as carbon dioxide or sodium sulfate [9]. Biogenic silica can replace the conventionally manufactured SiO_2_ in numerous applications from production of cement to drug delivery systems [6]. As morphology, particle size, structure, and purity of plant-based SiO_2_ depend on the processing parameters such as calcination temperature and time [7] as well as additional acid/base leaching [10], the properties of the final product can be finely tuned to specific applications, which widens the range of potential applications.

Silica is an important filler in the polymeric industry, which is mainly used as a reinforcement for rubber [11] as well as a filler for thermoplastics. Depending on the structure, silica can be a low-cost modifier of mechanical properties or a functional filler for nanocomposites tailored to be used in the production of sensors, optical devices, coatings, biomedical appliances, oil adsorbents, and more [12]. The biogenic form of this compound, especially rice husk ash (RHA), is also widely researched as a potential filler for thermoplastics. One of the first studies on the properties of RHA-filled polypropylene was conducted in 1993 by Fuad et al., who found that the addition of white and black rice husk ash improves the flexural modulus of the composites [13]. Since then, the influence of biogenic silica on different properties of a wide range of thermoplastic polymers has been evaluated. The polylactide (PLA)-based composites filled with plant-based SiO_2_ deserve special attention because of their sustainability, as well as their other promising properties.

Even though polylactide was synthesized for the first time in the 1930s by Carothers, it has gained popularity in the last 30 years as a more sustainable alternative to conventional polymers such as polypropylene or poly(ethylene terephthalate) [14]. In fact, PLA is synthesized from lactic acid, which is commonly produced during bacterial fermentation of starch originating from renewable sources such as corn or potatoes [15]. The currently available high-molecular-weight grades of polylactide can be processed using conventional methods such as extrusion, injection molding or 3D-printing; they can be also recycled and, at the end of life, subjected to industrial composting [16]. In addition to its environmental friendliness and good processability, this polymer is also characterized by advantageous mechanical properties such as high tensile modulus (≈4000 MPa) and tensile strength (≈60 MPa), so it can be applied in numerous industrial applications, including production of consumer products, mulching films for agriculture, automotive parts, packaging solutions, or even biomedical applications [15]. It should also be noted that mechanical and thermomechanical properties of PLA such as the stiffness, brittleness, or heat distortion temperature highly depend on the crystallinity of this polymer. Its crystalline phase can be characterized by more advantageous features (its Vicat softening temperature is about 100 °C higher in comparison with the amorphous PLA [17]), but, due to the slow crystallization rate of this polymer, high mold temperatures and long processing times are needed to obtain high crystallinity [18]. This is one of the factors which make the market share of this sustainable polymer still only a fraction of its fossil-based, non-compostable counterparts. Therefore, to widen the application potential of PLA, some research is still needed. The addition of modifying agents and fillers with nucleating ability is one way to achieve this goal. As can be read in the available literature, the application of fillers containing biogenic silica, such as rice husk ash, have a beneficial influence on the properties of this polymer. The main advantages, which are reported in the case of RHA-filled polylactide, are the improvement of stiffness [19], good barrier properties [19,20,21], higher thermal stability, and lower flammability [22]. Surprisingly, the application of biogenic silica originating from diverse sources apart from rice husk in polylactide composites has not been extensively researched. Also, the influence of SiO_2_-rich natural fillers on the crystallization of PLA has not been fully studied.

One of the most promising plants, which could be a resource of high-quality biogenic silica, is common horsetail (HT) (*Equisetum arvense* L.). It is a perennial plant, which is widely spread throughout the arctic and temperate regions of the Northern Hemisphere. Historically, horsetail has been applied in folk medicine as a treatment for various diseases including tuberculosis, as a hemostatic, and as a hair and nail growth enhancer [23]. It was also used as a polishing medium for pewter [24]. Nowadays, it is commonly assumed that HT’s effectiveness in these applications results from its chemical composition, especially from the high content of silica [24] and phenolic compounds [23,25]. Despite its wide availability and interesting composition, the application of horsetail in the polymeric industry has not been researched adequately. Even though there are a small number of papers discussing the addition of *Equisetum arvense* to natural rubber [11,26,27] or epoxy resin [28], its application in polylactide (or different thermoplastic polyesters) has not been described, to the best of our knowledge.

The subject of this paper is the influence of unmodified horsetail powder and HT-derived silica on the crystallization kinetics of polylactide. The process of crystalline phase formation in PLA is relatively slow and difficult, therefore a description of a novel filler’s influence on polylactide’s crystallization is interesting from both the industrial and scientific points of view. The obtained results will not only help to evaluate the application potential of HT and its silica as a filler for PLA, but also provide necessary information about the structure and polymer–filler interactions.

## 2. Materials and Methods

### 2.1. Materials

#### 2.1.1. Raw Materials

A multi-purpose polylactide grade Ingeo 2500HP produced by Nature Works (Minneapolis, MN, USA) was used as the matrix of composites. It is characterized by a melt flow index of 8 g/10 min (210 °C, 2.16 kg), a density of 1.24 g/cm^3^, a d-isomer content < 0.5%, a mass average molar mass of 193,250 Da, and a polydispersity index of 1.85. An extensive description of the characteristics of this PLA grade can be found in [29]. Horsetail sterile stems and leaves were harvested by the researchers in Chocz, Greater Poland area, Poland (51.98 N, 17.87 E). Analytical grade chloroform, sulfuric acid (96%), and sodium hydroxide were obtained from Avantor Materials Performance (Gliwice, Poland).

#### 2.1.2. Filler Preparation

Horsetail stems and leaves were dried at the air and ground using a Mockmill 200 Pro grain mill (Wolfgang Mock, Groβ-Umstadt, Germany). The ground horsetail was fractioned using an Analysette 3 (Fritsch, Idar-Oberstein, Germany) sieve shaker equipped with a 100 μm sieve.

The biogenic silica was extracted from the horsetail in a two-step procedure. First, acid leaching was performed to remove metallic impurities as well as holocellulose. Horsetail powder was introduced to 3.5% sulfuric acid solution in water and mixed at 80 °C for 3 h. After filtration and washing with distilled water, the solids, composed mostly of silica and lignin, were collected and dried overnight in the air. Then, calcination of the acid-purified HT was performed at 400 °C for 4 h using a muffle oven, and the obtained powder was ground in a ceramic mortar. A similar procedure was described by Battegazzore et al. in their paper on rice husk silica [19].

#### 2.1.3. Preparation of Composites

The composite samples were prepared using the solvent method. First, PLA and 1, 5, 10, or 25 wt% of the chosen filler were preliminarily mixed. A total of 1 g of the polymer–filler composition was introduced to 50 mL of chloroform and mixed at 55 °C for 1 h using a magnetic stirrer in a beaker closed with a lid. Then, the whole solution was poured into a 140 mm Petri dish. The film was first dried in the air for 24 h on a SK-0180-S laboratory shaker (DLAB, Beijing, China) set to 65 rpm, which prevented the agglomeration of the filler, and then at 55 °C for 24 h in a vacuum drier, to remove any solvent leftovers. Unfilled polylactide was prepared in the same way as the composites. The obtained samples were named in reference to their filler type and content. The films containing horsetail powder were marked as XXHT and the specimens with the biogenic silica were named XXSIL, where XX indicates the filler content in mass %.

### 2.2. Methods

#### 2.2.1. Filler Characterization

The size and distribution of the horsetail powder and silica particles was evaluated using an Analysette 22 laser particle sizer (Fritsch, Idar-Oberstein, Germany) in the range of 0.8–2000 μm.

The surface area and porosity of the fillers were determined using the Brunauer–Emmett–Teller (BET) method, using the ASAP 2420 apparatus (Micromeritics, Norcross, GA, USA). The nitrogen adsorption/desorption isotherms were performed at −195.15 °C on samples degassed at 120 °C for 12 h in a vacuum chamber. The specific surface area was determined by the multipoint BET method using adsorption data.

The crystallographic structure of the samples was analyzed by Empyrean X-ray diffraction equipment (Malvern Panalytical, Almelo, The Netherlands) with the copper anode (CuKα–1.54 Å) in the Bragg-Brentano reflection mode configuration with 45 kV and 40 mA parameters. The measurement parameters were set up for 3–60 with 45 s per step 0.05 in all cases.

Chemical composition of the fillers and the composites with 10 wt% of horsetail powder or silica was determined by Fourier-Transform Infrared Spectroscopy (FTIR) in the attenuated total reflection (ATR) mode using a FT/IR 4600 apparatus (Jasco, Tokyo, Japan). A total of 64 scans were performed with a resolution of 4 cm^−1^ in the range of 4000–500 cm^−1^.

#### 2.2.2. Non-Isothermal Crystallization

The process of non-isothermal crystallization was studied by the differential scanning calorimetry (DSC) method using a DSC 214 Nevio apparatus (Netzsch, Selb, Germany). First, samples of 5 ± 0.2 mg were placed in aluminum crucibles with pierced lids. The specimens were subjected to heating and cooling in the temperature range of 20–200 °C at a rate of 10 °C/min. The first heating was performed to equalize the thermal history of the samples and the second heating was analyzed. The obtained DSC thermograms were used to determine the values of the glass transition (*T_G_*), cold crystallization (*T_CC_*) and melting (*T_M_*) temperatures. The crystallinity degree *X_CR_* was calculated according to Equation (1).

(1) $$X_{CR}=\frac{∆H_{m}-∆H_{cc}}{(1-\varphi )\cdot ∆H_{PLA100\%}}\cdot 100\%$$

where Δ*H_m_* is the melting enthalpy of a sample, Δ*H_cc_* is the cold crystallization enthalpy of a sample, Δ*H_PLA_*_100*%*_ is the melting enthalpy of 100% crystalline PLA, Δ*H_PLA_*_100*%*_ = 93 J/g [30], and *φ* is filler content by weight.

#### 2.2.3. Isothermal Crystallization

Melt crystallization of PLA and its composites was also studied in isothermal conditions by the DSC method. The same apparatus, sample size, crucible type, and atmosphere as in the case of the non-isothermal measurements were used. The temperature program consisted of the following steps: heating from 20 °C to 200 °C at a rate of 50 °C/min, an isotherm for 5 min, cooling to the chosen crystallization temperature (100, 110, or 120 °C) at a rate of 150 °C/min, isothermal crystallization for 15 min, cooling to 20 °C at a rate of 50 °C/min, an isotherm for 5 min, and heating to 200 °C at a rate of 10 °C/min.

#### 2.2.4. Isothermal Crystallization Kinetics Evaluation

The kinetics of the isothermal crystallization were evaluated according to the Avrami method. According to this method, the crystallization of polymers can be described by Equation (2) [31]:

(2) $$1-X\left(t-t_{0}\right)=exp\left(-k{\left(t-t_{0}\right)}^{n}\right)$$

where *t* is time, *t*_0_ is the induction time, *X*(*t* − *t*_0_) is relative crystallinity at a time *t*, *k* is crystallization rate constant, and *n* is the Avrami exponent

Equation (2) can be rewritten to form (3):

(3) $$\ln\left[-\ln\left(1-X(t-t_{0})\right)\right]=n\ln\left(t-t_{0}\right)+\ln k$$

The values of the relative crystallinity *X*(*t* − *t*_0_) obtained from the DSC can be plotted as ln[−ln(1 − *X*(*t − t*_0_))] vs. ln(*t − t*_0_). The value *t*_0_ was determined as the beginning of the crystallization peak, i.e., the intersection of the crystallization curve and the baseline. The crystallization rate constant *k* can be then determined from the intercept of the straight line fitted to the experimental points and the Avrami exponent *n* is its slope. The calculations were conducted in the relative crystallinity range of 0.03–0.2, as advised by Lorenzo et al. [32]. The crystallization half-time *t*_1/2_*, i.e., the time needed to obtain *X*(*t* − *t*_0_) = 0.5 can be determined from the crystallization curves or calculated according to Formula (4):

(4) $${t_{1/2}}^{*}={\left(\ln1/k\right)}^{1/n}$$

#### 2.2.5. Microscopic Observations

The crystalline structure of polylactide and its composites was observed using polarized optical microscopy (POM). Samples were placed between two glass slides on a TMHS 600 hot plate (Linkam Scientific Instruments, Salfords, UK) and heated to 200 °C a rate of 30 °C/min. After a 2 min isotherm, they were cooled to 140 °C at a rate of 50 °C/min and held at this temperature for 30 min. The observations of the resulting crystalline structure were performed using an Eclipse E400 microscope (Nikon, Tokyo, Japan) equipped with a polarizer and an Opta Tech digital camera (Warsaw, Poland).

## 3. Results

### 3.1. Filler Characterization

#### 3.1.1. Particle Size Distribution

**Figure 1:**
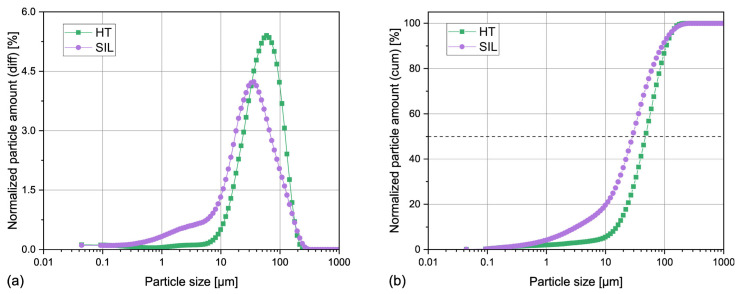
Differential (**a**) and cumulative (**b**) normalized particle amount of horsetail powder (HT) and biogenic silica (SIL).

The size distribution curves of the horsetail and silica particles are presented in Figure 1. The microscopic images of the filler can be found in the Supporting Information, in Figure S1. It can be observed that both filler types are characterized by a similar particle size, which is below 300 μm. In the case of HT, 90% of the particles are smaller than 110 μm and 50% do not exceed 48 μm. For the biogenic silica particles, it is 90 and 30 μm, respectively. This result is caused by a bigger population of smaller (<10 μm) particles present in the analyzed SIL powder. The microscopic observations confirm these results: most of the particles are smaller than 100 μm except for a few bigger clusters, which are more prominent in the case of the unmodified horsetail sample. The lack of large particles and aggregates in the SIL powder can be explained by its preparation procedure, especially the grinding of the calcinated powder.

#### 3.1.2. Surface Area and Porosity

**Figure 2:**
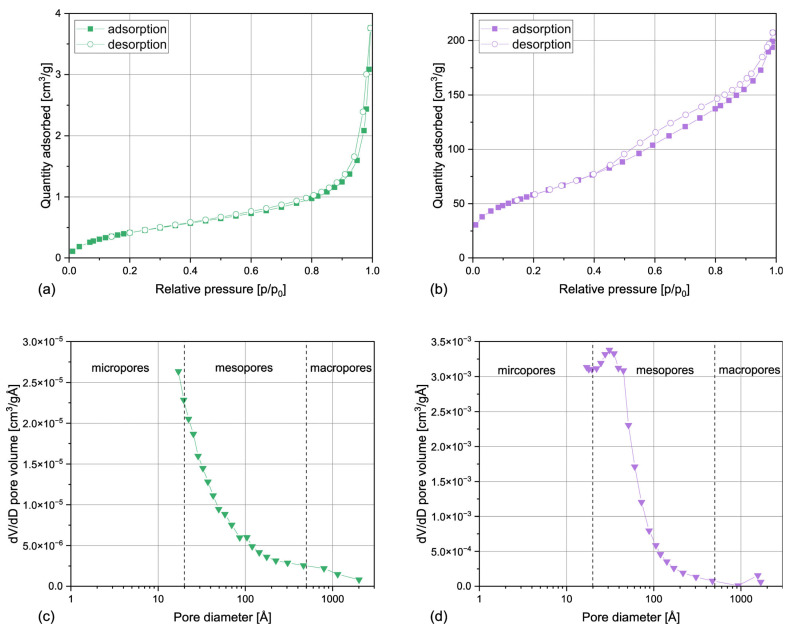
N_2_ adsorption/desorption isotherms obtained for horsetail powder (**a**) and silica (**b**); pore size distribution for horsetail powder (**c**) and silica (**d**).

The results of the surface area and porosity analysis, including BET surface area (S_BET_), BJH (Barrett–Joyner–Halenda) desorption cumulative surface area of pores (S_P_), and BHJ desorption average pore diameter (4V/A), are collected in Table 1. The nitrogen adsorption/desorption isotherms obtained for horsetail powder and silica are presented in Figure 2. As can be observed, both types of filler reveal entirely distinctive characteristics. The surface area of HT is relatively small and the obtained S_BET_ value is similar to the results also obtained for ground horsetail [33] or wood flour [34], so it can be considered a typical feature of a lignocellulosic material. The isotherm of this material can be classified as type II according to the IUPAC classification, which is typical for nonporous materials [35]. However, a very narrow H3-type hysteresis can be also seen in the case of HT, which indicates the presence of plate-like particles or macropores not completely filled with the condensate [36]. Despite the type-II isotherm, pores are present in the studied sample, which is also confirmed by the pore distribution plot. The BJH average pore diameter is about 120 Å and, as shown in Figure 2c, most of the pores present in HT can be classified as mesopores. Similar results have been described in the case of wood flour [34], so it can be stated that ground horsetail presents characteristics typical of a lignocellulosic material.

The silica obtained from horsetail presents completely different characteristics. It can be characterized by a large BET surface area of 214 m^2^/g. Interestingly, the increase in the surface area cannot be attributed to fragmentation of the particles during acid leaching, calcination, and grinding, because the laser particle analysis confirmed that HT and SIL samples can be characterized by a comparable particle size distribution (see Figure 1). Therefore, it can be stated that the shape of the horsetail powder was completely transformed during the silica production process. This claim is supported by the isotherm plot, shown in Figure 2b, which can be attributed to the IVa type with H1 hysteresis, according to the IUPAC classification [36]. The SIL powder can therefore be described as a mesoporous material consisting of cylindrical pores or spherical agglomerates [37], which presumably result from the spherical shape of silica particles present in *Equisetum arvense* tissues [24]. A similar shape of the nitrogen isotherm has been reported in the cases of horsetail ash [33] and amorphous silica from rice husk ashes [38]. Most pores present in the biogenic silica samples can be classified as mesopores with an average diameter of 56 Å. Interestingly, the pore distribution is similar in the case of both studied samples, but in the case of the silica, the pore population is shifted towards smaller diameters (<100 Å). It can be stated that SIL contains a higher number of smaller pores, hence the increase in specific surface area. Nevertheless, no pores smaller than 17 Å were detected in either sample. As similar results were also reported by Hosseini Mohtasham and Gholizadeh in the case of unmodified horsetail powder and horsetail ash [33], it can be presumed that this outcome results from the morphology of the phytolites present in the plant.

#### 3.1.3. Chemical Composition

**Figure 3:**
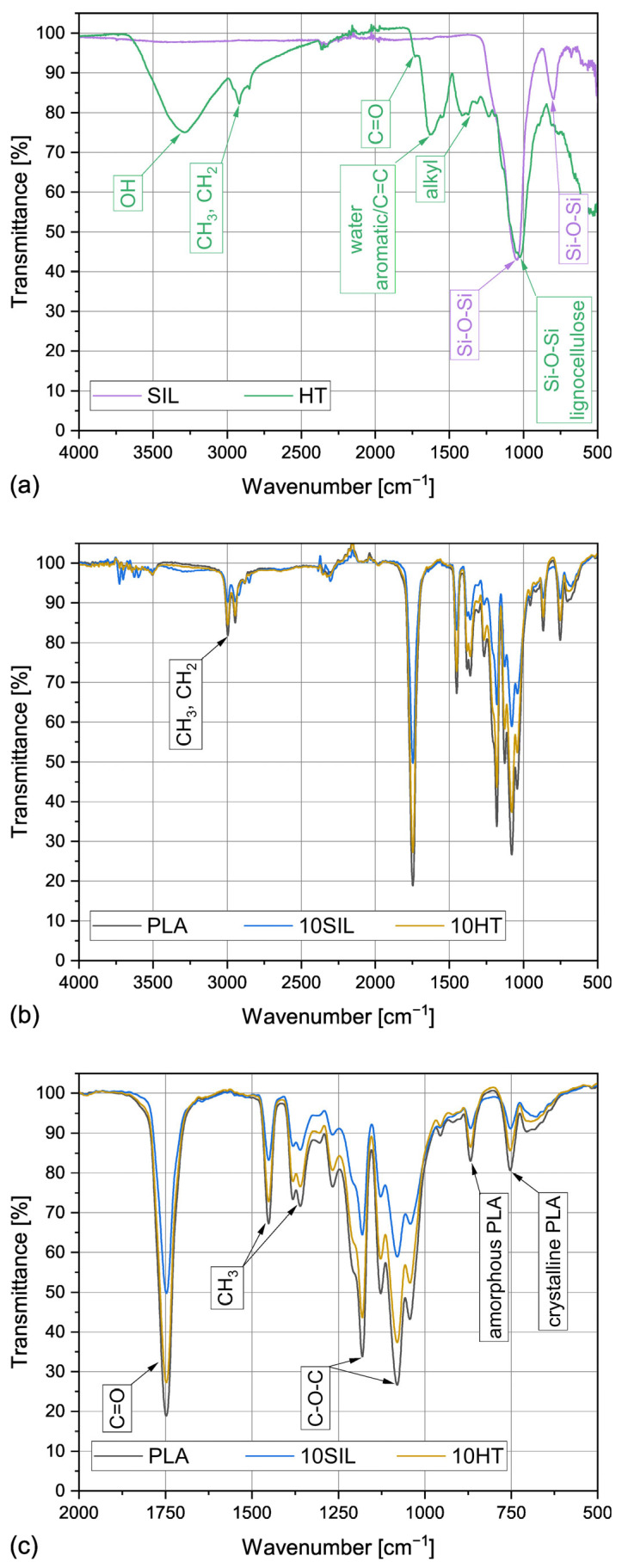
FTIR spectra obtained for horsetail and silica (**a**), PLA and its composites (**b**), and the enlarged 2000–500 cm^−1^ range of the PLA, 10SIL, and 10HT samples’ spectra (**c**).

The FTIR ATR spectra obtained for HT powder, silica, polylactide, and composites containing 10 wt% of each filler are shown in Figure 3. As can be seen, the silica prepared by acid leaching and calcination presents a composition typical for mineral SiO_2_, with only two prominent peaks at 1045 cm^−1^ and 796 cm^−1^ indicating the presence of Si-O-Si stretching [7,39], which implies high purity of the obtained biogenic filler. Horsetail powder presents a much more complex FTIR spectrum. A wide peak with its maximum around 3280 cm^−1^ comes from OH groups in water absorbed by this hydrophilic filler as well as bonded silanol groups [40]. Methyl (CH_3_) and methylene (CH_2_) groups of the lignocellulosic material can be observed at 2915 and 2850 cm^−1^ [26]. The peaks at 1416, 1374, and 1310 also indicate the presence of alkyl groups [40]. A small shoulder at about 1733 cm^−1^ results from the C=O vibrations of waxes, essential oils and resins present in the unmodified horsetail powder [40]. A more prominent peak at 1620 cm^−1^ can either indicate the presence of bonded water in the lignocellulosic component of HT or aromatic/C=C functional groups [26]. The most interesting feature of the horsetail powder’s spectrum are two overlapping peaks at 1046 and 1025 cm^−1^, which result from the presence of the lignocellulosic part of HT and the silica, respectively [7,26]. Therefore, it can be confirmed that horsetail powder is a hydrophilic, lignocellulosic filler containing considerable amounts of SiO_2_ as well as other substances such as essential oils or waxes.

The results of the chemical composition evaluation performed for PLA and the composites containing 10 wt% of biogenic silica or HT powder are presented in Figure 3b. Figure 3c shows an enlargement of the 2000–500 cm^−1^ region. The spectrum obtained for polylactide is typical for this polymer. Bands at 2995 and 2945 cm^−1^ result from stretching vibrations of C-H in CH_3_ and CH_2_ [41]. C-H bending in CH_3_ can also be seen as two overlapping peaks at 1381 and 1359 cm^−1^ as well as at 1451 cm^−1^ [41]. The 1747 cm^−1^ peak results from C=O stretching, and C-O-C valence vibrations of the aliphatic chains can be seen at 1180 and 1079 cm^−1^ [42]. The peaks at 867 and 753 cm^−1^ come from the amorphous and crystalline fractions of PLA, respectively [17]. Interestingly, the spectra of the composites filled with 10 wt% of HT or silica present an almost identical run to that of the unfilled PLA. The only difference is the intensity of the peaks. As the analysis was performed in the ATR mode, where the outermost layer of a material is studied, it can be stated that the filler particles are covered by a layer of the polymeric matrix. The decrease in the intensity of peaks, which is observed in the case of the composite samples, results from higher roughness of the samples’ surfaces and, consequently, the lower quality of the signal recorded by the apparatus. However, it can be concluded that the organic and inorganic filler particles, despite the differences in polarity of the polymer and the fillers, are well mixed with the polylactide matrix.

#### 3.1.4. Crystalline Structure

**Figure 4:**
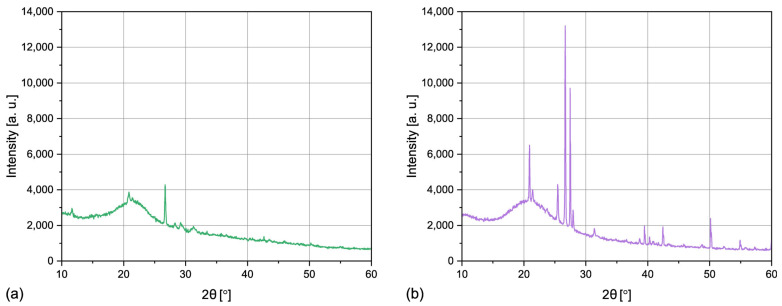
XRD patterns of horsetail powder (**a**) and the biogenic silica (**b**).

The X-ray diffraction patterns of horsetail powder and silica are presented in Figure 4. Both can be characterized by a wide peak centered at 22°, which can be attributed to amorphous silica [4]. This result indicates that SiO_2_ particles are in fact present in the stems of *Equisetum arvense*, which was also described by Holzhüter, Narayanan, and Gerber in their study [24]. It also confirms that the applied procedure of purification and calcination of the HT powder resulted in generation of biogenic silica. Moreover, the sharp peaks at 26° result from the presence of crystalline quartz [43]. According to the literature, acid leaching of the silicon-rich plant material followed by calcination below 600 °C should lead to formation of predominantly amorphous SiO_2_ [10,44]. In the presented case, the quartz particles present in the horsetail powder functioned as nucleation centers, which enabled the formation of the crystalline phase, despite the applied calcination temperature.

### 3.2. Crystallization of Composites

#### 3.2.1. Non-Isothermal Crystallization

**Figure 5:**
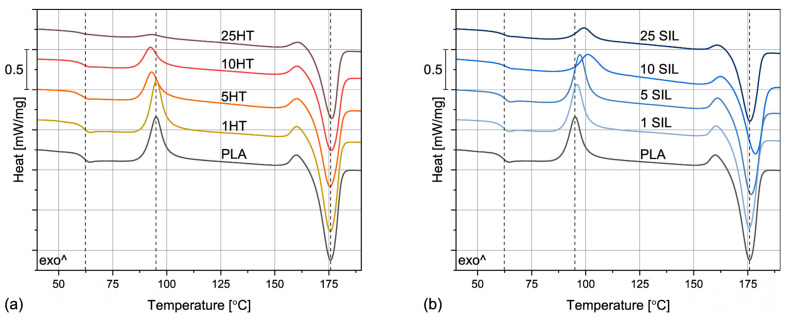
The DSC curves obtained during the second heating of PLA-based composites filled with horsetail powder (**a**) and biogenic silica (**b**).

The non-isothermal crystallization of PLA was studied by differential scanning calorimetry during heating and cooling at a rate of 10 °C/min. The thermograms obtained during the second heating are presented in Figure 5. The values of the glass transition (*T_G_*), cold crystallization (*T_CC_*), melting (*T_M_*), and melt crystallization (*T_CR_*) temperatures as well as the degree of crystallinity (*X_CR_*) are collected in Table 2. As can be observed, the run of the PLA’s thermogram is typical for the 2500HP grade, which contains <0.5% d-isomer and thus is able to form a crystalline structure. However, this process is not very efficient and the cooling rate of 10 °C/min is too high to allow for efficient crystallization, hence the cold crystallization peak present at 95.0 °C. The crystallinity degree of the unfilled polylactide is about 25.5%. Comparable results were obtained in our previous studies for the same grade of PLA subjected to melt processing [45]. Therefore, it can be stated that the solvent casting method did not alter the crystalline structure of this polymer and the solvent was removed from the sample.

The addition of both types of filler visibly changes the course of the DSC curves. The most noticeable difference can be observed in the case of the cold crystallization peak, which becomes less and less intense, especially for the samples with filler contents of 10 and 25 wt%. This trend is accompanied by the increase in crystallinity degree and appearance of melt crystallization peak, so it can be stated that both horsetail powder and silica have a positive impact on crystallization of PLA. Interestingly, even though the results obtained for the two fillers are similar, a lower content of HT is needed to induce melt crystallization and minimize the cold crystallization peaks. Another difference can be noticed in the case of glass transition temperatures—slightly lower *T_G_* values were determined for the samples filled with horsetail powder in comparison with the ones containing silica. This result could indicate presence of stronger interactions between the polymer and the SIL filler, whose particles, characterized by a large specific surface area, partially limit the possibilities of macromolecular movements. On the other hand, the melting behavior of all the studied samples, including the unfilled PLA, is comparable, so the composite materials can be processed in similar conditions to their matrix.

#### 3.2.2. Isothermal Crystallization

**Figure 6:**
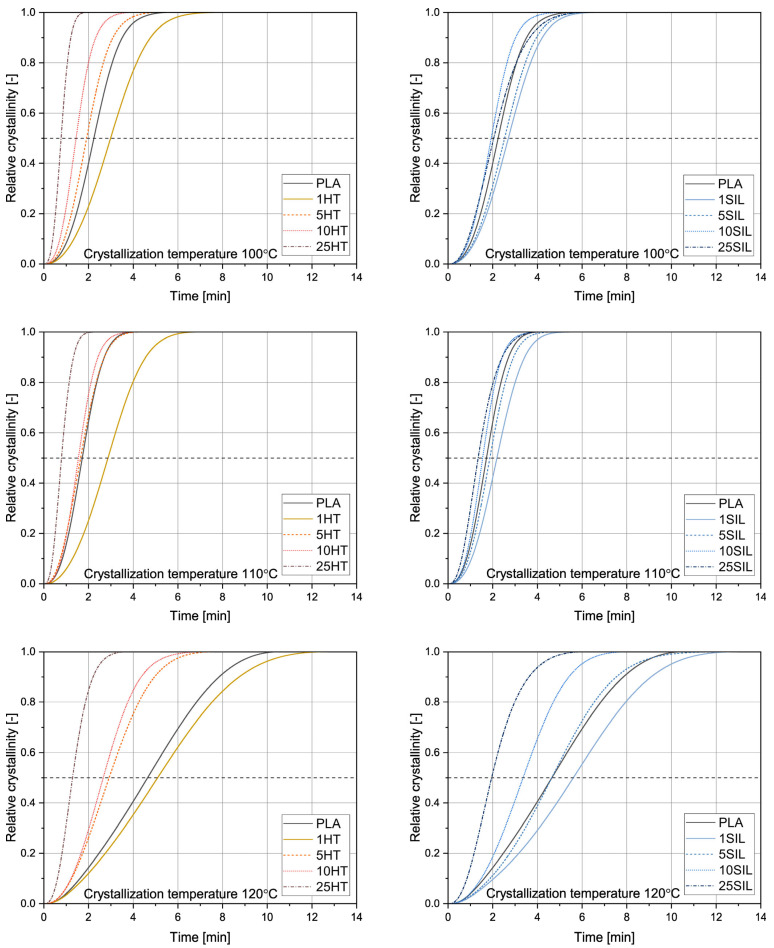
Isothermal crystallization curves obtained for HT- and SIL-filled polylactide composites at temperatures of 100, 110, or 120 °C.

**Figure 7:**
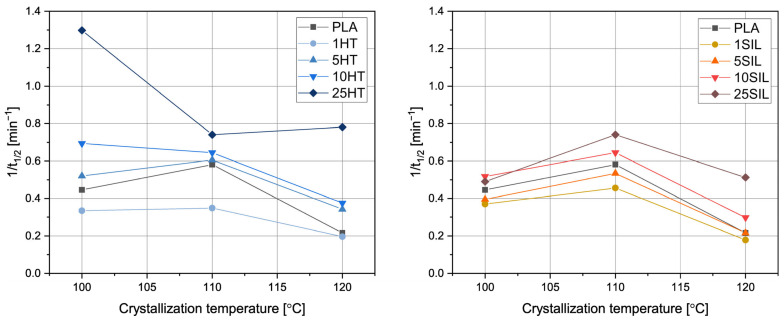
The inverse crystallization half-time as a function of the crystallization temperature determined for the PLA and its composites.

To further study the influence of horsetail-based fillers on crystallization of PLA, isothermal crystallization was performed at 100, 110, and 120 °C. The crystallization curves are presented in Figure 6. The logarithmic plots can be found in the Supplementary Information, in Figure S2. The values of crystallization half time obtained from the crystallization curves and calculated according to Equation (4) (*t*_1/2_ and *t*_1/2_*, respectively) along with the Avrami exponent *n* and crystallization rate *k* are collected in Table 3. The inverse of the crystallization half-time, which represents the overall crystallization rate [46], is shown in Figure 7 as a function of the crystallization temperature.

It can be observed that all the studied samples show the typical sigmoid crystallization curves. Based on their run, the *t*_1/2_ values were determined. For all the samples, regardless of the filler type and content, the shortest crystallization half-time (or, in other words, the highest overall crystallization rate represented by 1/t_1/2_) values were obtained at 110 °C, so this temperature can be considered close to the optimum (*T_opt_*). It is commonly reported that isothermal crystallization below *T_opt_* is hindered by the increasing viscosity of the melt (or the decreasing mobility of macromolecules), whereas at higher temperatures the supercooling is too low to provide suitable conditions for efficient nucleation [47]. A similar *T_opt_* value was determined by Blázquez-Blázquez et al. in the case of plasticized PLA filled with mesoporous silica [48]. For each crystallization temperature, a similar relationship between filler content and *t*_1/2_ can be seen. The samples containing 1 wt% of HT or SIL can be characterized by longer crystallization half-time than PLA. Interestingly, Hakim et al. found that addition of 3 wt% of surface-treated silica slightly reduced the crystallizability of polylactide-based blends because of strong interaction between the filler and the polymer, which reduced the possibilities of chain folding [49]. It can be noticed that the crystallization half-time of the remaining composites decreases with the amount of the added substance. Interestingly, Morales et al. studied the isothermal crystallization of polylactide filled with rice husk ash-based biosilica and obtained an inverse relationship between the SiO_2_ content and 1/t_1/2_ [50]. This difference can be attributed to the fact that biosilica was obtained in different conditions (calcination time and temperature) and can be characterized by divergent composition and morphology. Moreover, in our study, shorter crystallization half-times were noticed in the case of the composites filled with horsetail powder in comparison with their silica-filled counterparts. The *t*_1/2_ values of the HT-filled samples also change in a wider range, which suggests a stronger relationship between the filler content and its influence on PLA crystallization.

To further analyze the crystallization of PLA and its composites, the values of the Avrami exponent and crystallization rate constant were determined. R^2^ values above 0.9988 indicate that the chosen model is suitable to describe the crystallization of these materials, which is also confirmed by the similarity of the experimental and calculated values of the crystallization half-time. For the unfilled polylactide subjected to the isothermal crystallization at 110 °C (close to *T_opt_*), *n* is 2.55, which indicates heterogeneous nucleation with two-dimensional linear growth [31]. Similar values were obtained by Atanase, Glaied, and Riess in the case of PCL with different molecular build [46]. In fact, heterogeneous nucleation is commonly reported for polylactide, even when it is unfilled, where different kinds of impurities can serve as crystallization seeds [51]. The obtained *n* value is typical for isothermal crystallization of this polymer around 110 °C—for example, Wu et al. obtained an Avrami exponent of 2.52 in the case of PLA crystallized at 108 °C [52]. Crystallization at both higher and lower temperatures causes a decrease of *n*—at 120 °C it does not exceed 2, so the growth of the crystallites changes from two- to one-dimensional. Addition of 1 wt% of either kind of filler also results in a slight decrease of *n*, but the Avrami exponent then increases with the filler content. This growth is especially noticeable in the case of crystallization at 120 °C, when the 25HT and 25SIL samples achieve Avrami exponents of 2.63 and 2.39, respectively. It can be stated that addition of both fillers allows the favorable two-dimensional growth of crystallites to be retained despite the disadvantageous thermodynamical conditions. Similar results were obtained for polylactide with 3 wt% of hydrophilic nano-silica, of which the *n* value at 120 °C was increased from 2.20 to 3.23 [53]. Presumably, the presence of the filler particles causes local disturbances of temperature, so the supercooling in the vicinity of the filler particles is higher in comparison with the bulk polymer.

The obtained values of crystallization rate constant also reflect the influence of the filler type and amount on the formation of the ordered phase. Most notably, the *k* value of the 25HT sample crystallized at 110 °C is one order of magnitude higher in comparison with the unfilled PLA. This constant follows the trend also shown by *t*_1/2_ and *n*—the most favorable results are obtained by highly filled composites subjected to crystallization at a temperature close to *T_opt_*.

When analyzing the crystallization kinetics parameters of PLA and its composites, it can be noticed that some issues need a more thorough explanation. First, why does the unmodified horsetail powder have a more beneficial influence on the formation of the polylactide crystallites than silica? This question can be answered when the rigid amorphous fraction (RAF) is considered. According to Papadopoulos et al., RAF can be found in polymeric nanocomposites, where it can be defined as the interfacial bound polymer attached to the filler nanoparticles [54]. In the case of nanocomposites based on semicrystalline polymers, an additional rigid amorphous fraction around the crystallites can also be found. The bound polymer can either facilitate or make the crystallization of the matrix more difficult. Depending on the size of the filler particles, RAF can be merged with the crystallites; otherwise, when the added particles form large aggregates, the bound polymer around them remains in the amorphous phase [55]. As has been was experimentally shown in the case of polylactide-based composites with carbon nanotubes and montmorillonite clay, stronger interactions between the filler and the polymer (or a larger content of RAF around filler particles) limit the substance’s ability to improve the crystallization [54]. An analogous situation takes place in the described case. When the biogenic silica, which is characterized by a more prominent population of small particles and considerably larger specific surface area than horsetail powder, is introduced to polylactide, its macromolecules have more possibilities to interact with the filler particles and form RAF around them. Because of the relatively large size of the SIL particles (in comparison with the nanocomposites described in the literature), they cannot be embedded inside the crystallites and remain in the amorphous phase. Therefore, even though addition of biogenic silica can have a positive impact on PLA crystallization, it is limited by creation of RAF around its particles. On the other hand, weaker interactions between the polymeric matrix and the horsetail powder particles may facilitate the rearrangement of the chains during crystallization, but also decrease the mechanical properties of the composite, such as crack resistance.

On the other hand, the proposed description based on the formation of a rigid amorphous fraction around the filler particles does not explain why addition of HT and SIL, especially at content of 10 wt% or more, facilitates crystallization of PLA at all. In fact, the polymer–filler interactions are not the only factor influencing the process of formation of the ordered phase. Supercooling is also a crucial parameter, so the differences between the samples are the most noticeable in the case of isothermal crystallization at 120 °C, when the difference between the melting temperature and the crystallization temperature is too small to create a sufficient number of self-nuclei [54]. Therefore, incorporation of both SIL and HT provides additional heterogeneous seeds of crystallization and causes local increases in supercooling so the two-dimensional growth of crystallites can take place.

Another factor which affects the filler’s influence on the crystallization of a polymer is its distribution. When 1 wt% of SIL or HT is added to polylactide, the filler particles are distant from each other, so they only influence the crystallization and formation of RAF in their closest proximity. As filler content increases, its particles become more densely distributed and their impact on bulk crystallization of PLA is more visible. Because of this, both the *n* and *k* values as well as the crystallinity degree increase with filler content.

**Figure 8:**
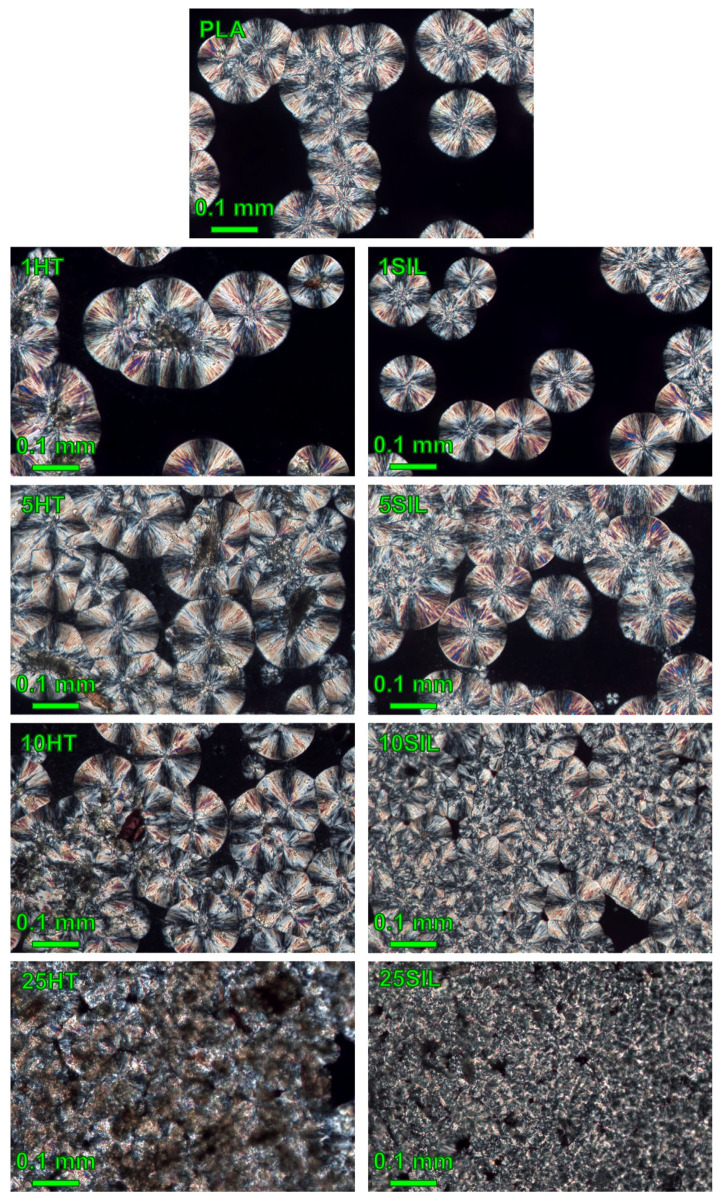
Microscopic images of the samples after isothermal crystallization at 140 °C.

To confirm the results of the isothermal crystallization kinetics analysis, microscopic observations of the structures of the samples were performed in polarized light. The obtained images are shown in Figure 8. It can be observed that the chosen isothermal crystallization temperature of 140 °C allowed for obtaining large crystallites, which are easy to observe. In the case of the unfilled polylactide, uniform-sized spherulites scattered in the amorphous phase can be seen. Because supercooling at this temperature is low, the growth rate of crystallites is higher than the rate of deposition of nuclei, so the spherulites are rarely in contact with one another. The addition of 1 wt% of horsetail powder does not change the density of the crystallites, but they become larger. This change is caused by the fact that filler particles are situated at the center of the spherulites, so their diameter needs to increase accordingly. It can be concluded that the HT particles are the true crystallization seeds for PLA. It should be noted that the structure of the 1SIL composite is different—in this case, the spherulites are slightly smaller in comparison with PLA and filler particles are visible in the obtained image. It can be presumed that silica particles are situated in the amorphous phase where, because of the presence of RAF, they limit the growth of spherulites. Nevertheless, this crystallization regime also takes place in the case of both composites containing 1 wt% of the filler.

An increase in filler content also results in a growing density of spherulite distribution. At filler contents of 5 and 10 wt%, the crystallites start to impinge on one another, covering almost the entire observed area. The growth rate of the spherulites is comparable with the rate of nucleation. However, the distribution of the crystallites in the field of view is not uniform—regions densely packed with crystallites are accompanied by amorphous areas. This may result from the non-uniform width of the studied samples cut from the solution-cast films. In the case of the 25HT and 25SIL samples, almost no amorphous phase is visible in the field of view and the spherulites become so fine that it is hard to distinguish the individual crystallites. When we compare the two types of composite with the filler percentage of 5 wt% or more, it is clearly visible that the horsetail powder-filled samples create larger spherulites containing a filler particle at their center, whereas application of silica results in finer structure. As no filler particles are visible in this case, it may be assumed that they are not “true” crystallization seeds, but are rather located in the amorphous phase and influence the crystallization of PLA by changing local temperature distribution and causing local flow disturbances.

## 4. Conclusions

Biogenic silica was successfully obtained from horsetail powder. It was characterized by a large specific surface area of 214 m^2^/g, a mean particle size of 30 μm, and a mesoporous structure. Despite acid leaching and a calcination temperature of 400 °C, a crystalline phase was present in the structure of silica, which was caused by the presence of quartz crystallites in the horsetail powder. It was confirmed that *Equisetum arvense*, a widely spread plant, can be a source of high-quality biogenic silica, which can be utilized in the polymeric industry.

The influence of horsetail powder and biogenic silica on crystallization of polylactide was determined. It was found that the addition of 25 wt% of HT or SIL increases the crystallinity of the polymer by 100%. The presence of both fillers also reduces the crystallization half-time from 4.6 min to 2.0 or even 1.3 min (in the case of the 25SIL and 25HT samples, respectively) at 120 °C when the supercooling is too low to make the nucleation and growth of crystallites efficient. Even though the two studied fillers have a similar influence on PLA crystallization kinetics parameters such as the crystallization rate constant *k*, which increased with the filler content, the mechanism of their action is different. As was shown by the optical microscopy in polarized light, the horsetail particles serve as nucleation seeds for polylactide and induce the creation of large crystallites with the filler at their center. On the other hand, because of their larger specific surface area, the silica particles are surrounded with a layer of bound polymer—the so-called rigid amorphous fraction—and therefore remain in the amorphous phase of the composite.

Apart from the mechanism of action, it can be decided that both filler types have a beneficial influence on crystallization of PLA. Horsetail, a ubiquitous and low-demand plant, can be applied in the polymer processing industry as a plant-based filler to shorten the processing time and improve the efficiency of production of consumer and structural parts by the injection molding method.

## Figures and Tables

**Table 1:** Surface area and porosity of horsetail powder and biogenic silica.

Sample	S_BET_	S_P_	4V/A
	m^2^/g	m^2^/g	Å
HT	2	2	120
SIL	214	241	56

**Table 2:** The results of non-isothermal crystallization analysis by DSC.

Sample	*T_G_* [°C]	*T_CC_* [°C]	*T_M_* [°C]	*T_CR_* [°C]	*X_CR_* [%]
PLA	62.3	95.0	175.8	-	25.5
1HT	61.8	95.2	175.7	-	22.9
5HT	61.3	93.0	175.6	99.1	34.9
10HT	61.4	92.6	176.2	97.6	38.2
25HT	62.4	92.9	176.3	99.4	52.5
1SIL	62.4	95.9	175.7	-	22.7
5SIL	63.1	97.2	176.4	-	25.8
10SIL	63.4	101.1	178.4	99.2	41.2
25SIL	63.1	99.1	175.9	103.2	51.1

**Table 3:** The results of the isothermal crystallization kinetics analysis.

Temperature	Sample	*t*_1/2_ [min]	*t*_1/2_* [min]	*n* [-]	*k* [min^−*n*^]	R^2^
100 °C	PLA	2.24	2.31	2.48	0.087	0.99997
	1HT	2.99	3.08	2.28	0.053	0.99999
	5HT	1.92	1.92	2.55	0.131	0.99998
	10HT	1.44	1.45	2.67	0.258	0.99999
	25HT	0.77	0.77	2.86	1.462	0.99999
	1SIL	2.70	2.79	2.38	0.060	0.99997
	5SIL	2.53	2.63	2.40	0.068	0.99994
	10SIL	1.93	1.97	2.57	0.122	0.99998
	25SIL	2.04	1.97	2.40	0.137	0.99988
110 °C	PLA	1.72	1.74	2.55	0.169	0.99999
	1HT	2.87	2.95	2.26	0.060	0.99999
	5HT	1.65	1.61	2.42	0.219	0.99991
	10HT	1.55	1.55	2.73	0.209	0.99999
	25HT	0.79	0.76	2.79	1.503	0.99993
	1SIL	2.19	2.22	2.51	0.094	0.99999
	5SIL	1.87	1.87	2.59	0.134	0.99999
	10SIL	1.55	1.55	2.73	0.209	0.99999
	25SIL	1.35	1.28	2.53	0.369	0.99987
120 °C	PLA	4.64	4.41	1.90	0.041	0.99879
	1HT	5.09	5.09	1.81	0.036	0.99946
	5HT	2.92	2.85	2.27	0.064	0.99992
	10HT	2.66	2.65	2.43	0.065	0.99998
	25HT	1.28	1.25	2.63	0.389	0.99997
	1SIL	5.61	5.99	1.77	0.029	0.99958
	5SIL	4.64	4.96	1.94	0.031	0.99997
	10SIL	3.36	3.42	2.26	0.043	0.99999
	25SIL	1.95	1.86	2.39	0.157	0.99989

## Data Availability

Dataset available on request from the authors.

## Supplementary Materials

The following supporting information can be downloaded at: https://www.mdpi.com/article/10.3390/ma17235697/s1, Figure S1: Microscopic image of horsetail powder (a) and the biogenic silica (b); Figure S2: Logarithmic plots of the crystallization curves.
