# Supplementary figures and images for: The Influence of Horsetail (Equisetum arvense L.) Powder and Horsetail-Based Silica on the Crystallization Kinetics of Polylactide
Source: Materials (Basel). 2024 Nov 21;17(23):5697. doi: 10.3390/ma17235697 (PMC11641935; doi:10.3390/ma17235697)

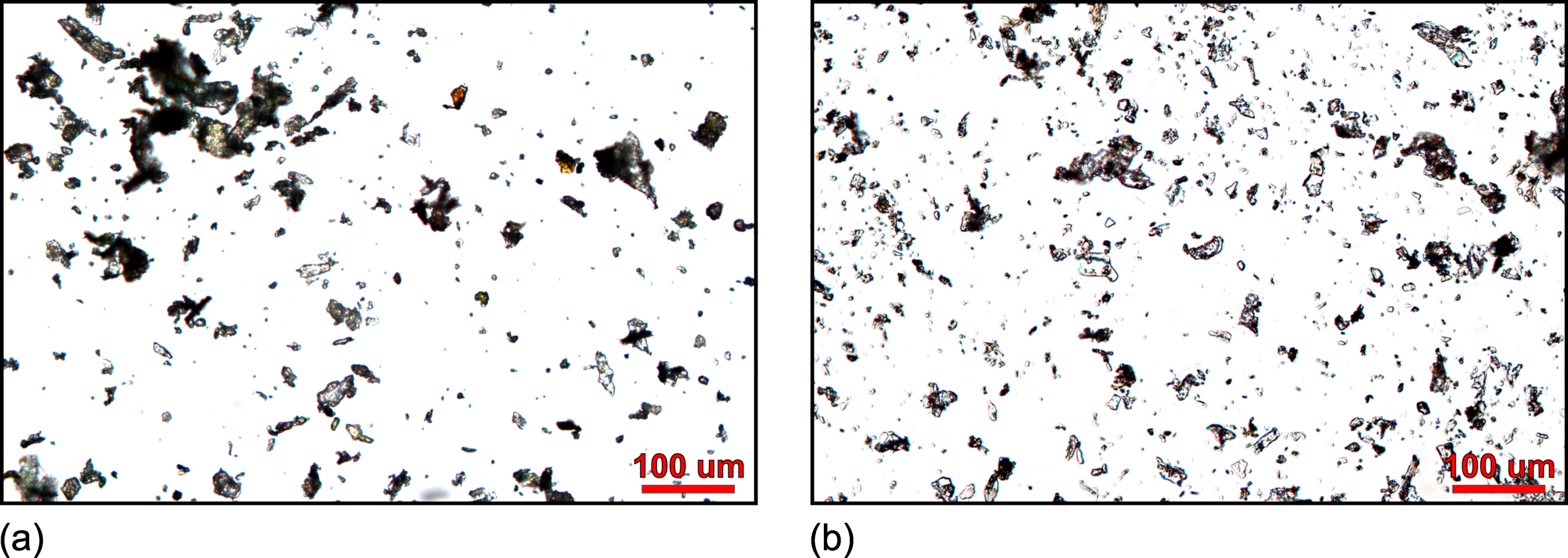

Supplement: Supplementary file 1 [file materials-17-05697-s001.zip › Figure S1.tif]

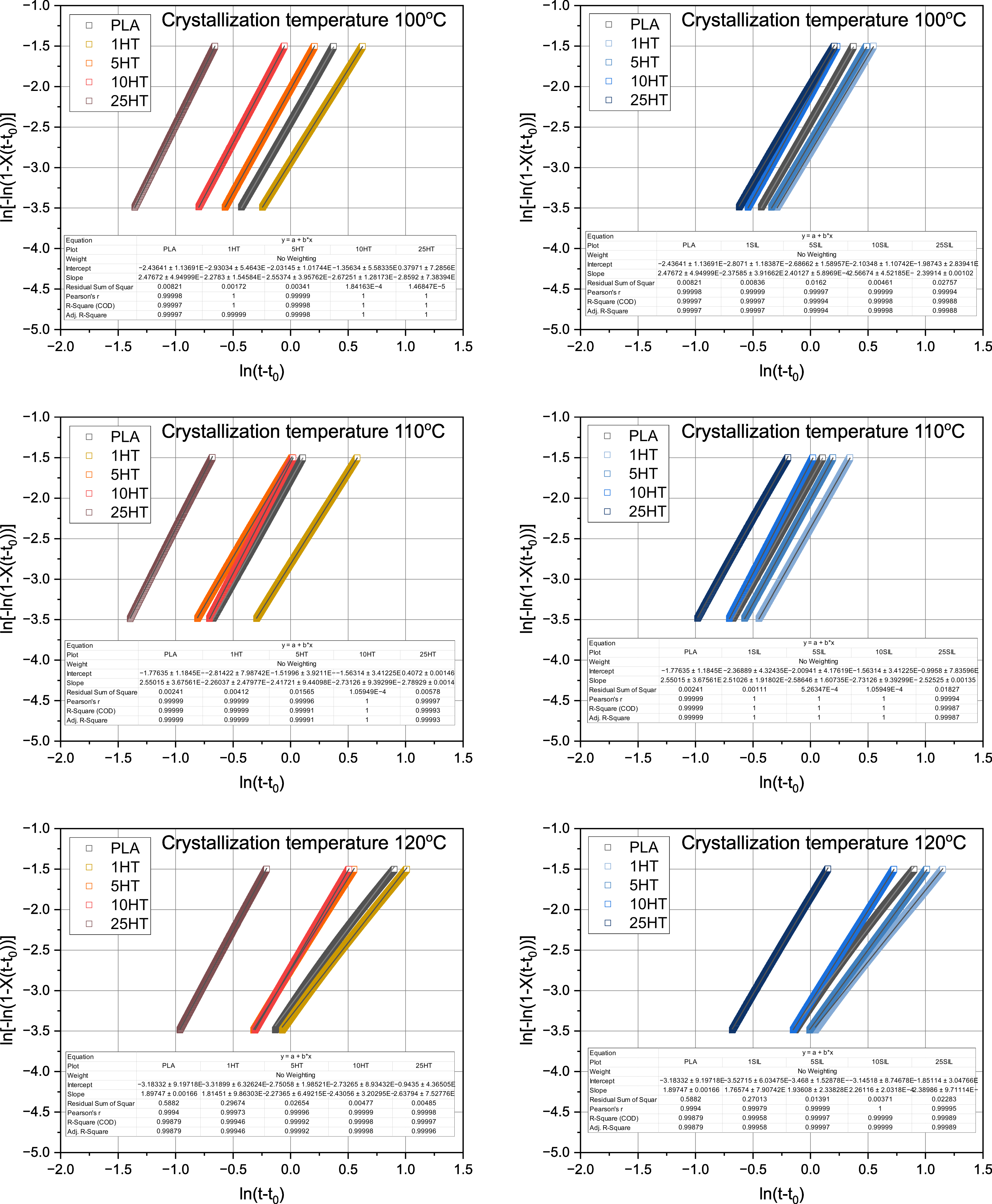

Supplement: Supplementary file 1 [file materials-17-05697-s001.zip › Figure S2.tif]
